# Toxicodynamics of Mycotoxins in the Framework of Food Risk Assessment—An In Silico Perspective

**DOI:** 10.3390/toxins10020052

**Published:** 2018-01-23

**Authors:** Luca Dellafiora, Chiara Dall’Asta, Gianni Galaverna

**Affiliations:** Department of Food and Drug, University of Parma, 43124 Parma, Italy; luca.dellafiora@unipr.it (L.D.); chiara.dallasta@unipr.it (C.D.)

**Keywords:** mycotoxins, toxicology, in silico molecular modeling, toxicodynamic, risk assessment computational toxicology, mechanism of action

## Abstract

Mycotoxins severely threaten the health of humans and animals. For this reason, many countries have enforced regulations and recommendations to reduce the dietary exposure. However, even though regulatory actions must be based on solid scientific knowledge, many aspects of their toxicological activity are still poorly understood. In particular, deepening knowledge on the primal molecular events triggering the toxic stimulus may be relevant to better understand the mechanisms of action of mycotoxins. The present work presents the use of in silico approaches in studying the mycotoxins toxicodynamics, and discusses how they may contribute in widening the background of knowledge. A particular emphasis has been posed on the methods accounting the molecular initiating events of toxic action. In more details, the key concepts and challenges of mycotoxins toxicology have been introduced. Then, topical case studies have been presented and some possible practical implementations of studying mycotoxins toxicodynamics have been discussed.

## 1. Introduction: Food Toxicology and Risk Assessment

Food toxicology defines the science dealing with the toxicological effects of food constituents [1]. Bearing in mind the old-date assumption “*let thy food be thy medicine and thy medicine be thy food*” (attributed citation; Hippocrates of Kos at the turn of the fourth and the fifth century BC), the role of diet in keeping the health and wellbeing of humans and animals has been scientifically proved today. Indeed, food and feed are among the most complex cocktails of low-molecular weight xenobiotics to which the living organisms are exposed [2]. This multiple (i.e., various chemo-types are involved), combined (i.e., different chemo-types can be combined together) and in some cases prolonged exposure (i.e., chronic exposures, even with extended time-course, are possible) may have severe impairing effects on humans and animals [3]. Extensive considerations on the nature and the route of food and feed contamination would be out of the scope here (detailed information can be found in [4]). However, it is worthy to quote that the naturally occurring contaminants, i.e., the unwanted molecules accumulating in food and feed produced by living organisms, are of major concern because they are typically hard to control (even unavoidable in some cases) and manage [5]. Among them, mycotoxins have been object of a growing number of investigations in the last years as they may severely threaten human and animal health [6].

The overall burden of mycotoxins encompasses medical costs, financial detriments and food waste [7,8]. Therefore, many countries have adopted regulations to limit the overall mycotoxin dietary exposure, safeguarding the consumers and animal health. At the same time, the setting of regulatory actions must pursue tricky tradeoffs to avoid unreasonable food wasting and to limit trade frictions [9]. In order to address such a premise, the regulatory actions have to rely on sound risk assessment studies that must be based on a solid background of toxicological knowledge. So far, risk assessments studies have been carried out on some major mycotoxins from the public health perspective (i.e., aflatoxins, fumonisins, ochratoxin A, deoxynivalenol and zearalenone) by many international agencies and organizations (including the European Food Safety Authority (EFSA)) to set appropriate food safety regulation and recommendations. Obviously, the deeper the toxicological understanding, the more appropriate, precise and effective the risk assessment of a molecule (or a class of molecules) can be. In this regard, however, many aspects in the toxicology of mycotoxins are not fully understood yet, especially from a mechanistic point of view. This ultimately poses a degree of uncertainty to the current risk statements [10]. In addition, beside the few mycotoxins regulated so far, many more can contaminate food and feed. They are referred to as “emerging mycotoxins” and include a great variety of chemo-types. Alternariol and alternariol methyl ether, enniatins, moniliformin, beauvaricin, culmorin and fusaric acid are among those gathering wide attention [11]. However, the shortage of toxicological data and the lack of a convincing consensus on toxicity and on the mechanisms of action prevent from setting appropriate guidelines.

Overall, this scenario points out the need to move further steps in understanding mycotoxins toxicology to timely fill the data gaps and, therefore, to provide a sufficiently informed scenario for risk assessment.

Keeping in mind that the full comprehension of mycotoxins toxicology is a major challenge for the Scientific Community and it requires inter-disciplinary approaches, this work focuses on the possible support offered by computational methods in the field of toxicodynamics, moving from principles and potentialities (Section 2 and Section 3) to the application to case-studies (Section 4) and outlooks (Section 5).

## 2. Toxicokinetics and Toxicodynamics in Chemicals Risk Assessment

Risk assessment is referred to as the stepwise and iterative process aimed at estimating from qualitative or quantitative point of view the likelihood, or the magnitude and probability, of harmful effects to individuals or population due to a given chemical or group of chemicals. Briefly, the main steps in chemical risk assessment are the following [12]: (i) Hazard identification, which is the identification of compounds (“hazard”) able to cause adverse effects; (ii) hazard characterization, which allows the description of the adverse effect in terms of dose-response and mode of action; (iii) exposure assessment, that is the analysis estimating the intensity, the frequency and the duration of the exposure to a given chemical; (iv) risk characterization, which combines the information of exposure and toxicity to qualitatively/quantitatively estimate whether an individual or a population is exposed to a risk.

Typically, in the chemicals risk assessment, the hazard identification and hazard characterization rely on extrapolations from toxicological data resulting from in vitro or, better, in vivo animal studies. However, understanding the relationship between the mechanistic aspects of toxic action and the time-dependency of toxicity is an undeniable prerequisite to undergo meaningful risk assessment studies [13]. This relationship is typically explained from a toxicokinetics and toxicodynamics perspective.

### 2.1. Toxicokinetics

Toxicokinetics determines the relationship between the concentration of a given chemical to which an individual is exposed, and the concentration of toxicologically active compounds at the district of action, intended as the organ, tissue, cellular or molecular locus where the chemicals exert the effect. While multiple routes of exposure may exist for mycotoxins (e.g., skin absorption and inhalation [14,15]), the present work focuses on the dietary intake as considered the prevalent route so far [7]. Therefore, in this case, the differences between the ingested concentration and the concentration at the site of action are explained by the LADME paradigm [16]. In more detail, the intensity of the effect of any food toxicant primarily depends on its bioaccessibility, that determines the fraction of toxicants that is released from the food matrix in the gastrointestinal tract upon digestion, becoming thus available for the absorption (L = liberation). Then, a fraction of the released toxicants is absorbed (A = absorption) reaching the bloodstream by which they may be distributed into the body tissues (D = distribution). In the meanwhile, the absorbed compounds undergo metabolism resulting in a multitude of metabolites (M = metabolism) at both pre-systemic and systemic level until they are excreted via renal or biliary processes (E = excretion). While dietary habits are crucial for the definition of the exposure to food contaminants, the persistence of toxicologically active compounds in the living organisms depends on the ADME kinetics and eventual bioaccumulation phenomena [6].

### 2.2. Toxicodynamics

Toxicodynamics determines the relationship between the concentration of a toxicant at the site of action and the toxic effect at the level of molecule, cell, tissue, organ or organism. According to the Druckrey–Küpfmüller model [17]—which well describes the dynamics of non-genotoxic compounds with a specific mode of action [13]—the dynamics of toxic action relies on the interaction between the toxicant and specific molecular target(s), referred to as the “receptor(s)”. It must be noticed that, in the context of host–guest complex formation, the term “receptor” is commonly used to indicate the host (i.e., the biological target) recognized by the guest (i.e., the mycotoxins) regardless of its biological role (i.e., whether it is an enzyme, a carrier or a receptor in the strict sense) [18,19]. Typically, the receptors are proteins, but they can be also other macromolecules such as DNA- or RNA-protein complexes and glycoproteins. The receptor binding is the primal molecular event of non-genotoxic compounds action determining the so defined molecular initiating event (MIE) [20]. The receptor binding is causally connected to the toxic effects through a cascade of further molecular interactions that comprehensively defines the “mechanism of action” of a given compound [21]. In turn, this cascade of molecular events determines a series of functional and structural modifications at the level of cells, tissue or organs, that is chorally referred to as the “mode of action” (MoA) [22]. Each causally connected event from the receptor binding to the final adverse outcome is linearly connected defining the adverse outcome pathway (AOP) [23].

Concerning the early step of MIE, non-genotoxic compounds establish a dynamic binding with the receptors reaching the steady-state equilibrium in a given time frame, depending on the kinetic of association and dissociation [24]. From a molecular point of view, this binding event typically determines structural changes altering the normal receptor structure and functionality, and leading thus to perturbation of homeostasis. The toxic outcome onsets when such perturbation exceeds a threshold that cannot be tolerated. The relative concentration of the bound receptor at the site of action strongly influences the intensity in triggering the toxic stimulus. In turn, the relative concentration of bound receptor depends on the concentration of toxicant at the site of action, its binding affinity and the kinetics of association and dissociation. On this basis, it can be argued that the strength of interaction (namely, the thermodynamic favor of the ligand-receptor complex formation) can proportionally correlate to the potency in triggering the early step of toxic stimulus. Therefore, in the context of the chemical risk assessment process, toxicodynamics investigations focused on the receptor binding may support both steps of hazard identification and characterization by probing the primal molecular event of toxic action (*vide infra*).

For many years, the toxicological understanding of mycotoxins (including the dynamics aspects) has been based mainly on the independent assessment of few reference molecules representing broad classes of compounds (i.e., zearalenone has been considered for a long time the reference for all its fungal and plant analogues). Nowadays, however, food myco-toxicologists and risk assessors are moving toward a group-based approach to better address the multi-toxins exposure paradigm (e.g., [25,26]). In this framework, the risk related to mycotoxins exposure should be assessed considering the combined action of multiple compounds considering either the simultaneous exposure to different chemo-types (i.e., mycotoxins belonging to different classes) and the exposure to multiple analogues of a given chemo-type (e.g., the metabolites originated from a specific parent compound) [27]. Indeed, food and feed are typically a source of multiple toxins as they may contain not only different mycotoxins chemo-types, but also many metabolites for each class of toxin. Even though most of these metabolites and analogues are still lacking of a sufficient toxicological characterization, some of them have proved to be stronger than the parent compounds in triggering the molecular cascade underlying the AOP. As an example, keeping in mind that some members of the zearalenone group bind and activate the estrogen receptors, α-zearalenol was proved able to bind the estrogen receptors more strongly than the parent compound zearalenone [28,29]. Therefore, it can be argued that the relative abundance of the differently active metabolites at the site of action may have a relevant role in determining the magnitude of toxic stimulus triggering, with possible consequences also on the magnitude of the overall toxic effect. On this basis, the toxicodynamics of mycotoxins may strongly depend on the overall concentration at the site of action of all the mycotoxins metabolites and analogues that are able to bind the receptor. Hence, the investigation of mycotoxins toxicodynamics should be amended dismissing the bimolecular paradigm (one molecule-one receptor) and adopting the principle of “toxic equivalents”. Contextually, toxic equivalents are referred to as those molecules that may occur at the site of action and may act as functional analogues in binding a specific receptor. Accordingly, the understanding of the dynamic aspects of mycotoxins toxicity ideally requires a comprehensive assessment of all the toxic equivalents that can be found at the site of action. Following this principle, EFSA has recently provided health-based guidelines for zearalenone considering the relative potency factors of the main modified forms [26].

Extending such approach to the other mycotoxins poses a major challenge. Indeed, the relative contribution of each toxic analogue cannot be easily extrapolated from the in vitro and in vivo toxicological data. Moreover, most of mycotoxins metabolites and analogues are not available on the market making extremely cost-effective and labor-intensive their comprehensive assessment using canonical experimental trials [30].

## 3. In Silico Analysis in Risk Assessment: A Toxicodynamics Perspective

Keeping in mind that both kinetic and dynamic aspects are relevant to fully understand the toxicity of mycotoxins, computational methods may model both the toxicodynamics and toxicokinetics of small molecules [31,32,33]. However, the present work focuses only on the dynamic aspects, deepening how the in silico analysis of the primal molecular event underlying the toxic action might have a role in advancing knowledge in mycotoxins toxicology. Considering the complexity of the toxicological actions and the different aspects taken into account by the risk assessment process (see Section 2), the assessment of the mycotoxins-related risks inevitably requires an inter-disciplinary approach where molecular and cellular insights integrate in vivo evidences. In such a complex scenario, the approaches proposed in this work address the molecular aspects and, in particular, they deepen the toxicant-receptors interplay at the very beginning of the molecular cascade of the toxic action. Therefore, this kind of study may expand the understanding of the primal mechanism of action of toxicants and they may integrate and support the experimental toxicological investigation during the early phases of risk assessment (i.e., hazard identification and characterization).

Historically, the computational analysis of molecules bioactivity (including toxicity) has relied on ligand-based and structure-based approaches [34,35,36]. In particular, ligand-based approaches are typically based on the structures of molecules neglecting any direct information on the receptors structure and nature. They have been developed for the first time in the early 1960s, when attempts to correlate the structures of molecules to the biological activity were made using the first computers [37]. These methods are based on the general assumption that the biological properties of small molecules (from the capability to be absorbed in the gastrointestinal tract to the triggering of toxic stimulus *via* receptor binding) are function of their chemical structure [38,39]. Indeed, any chemical structure can be described by combinations of the so defined molecular descriptors that, ideally, are based on measurable physicochemical properties. The appropriate sorting of these descriptors may correlate qualitatively or quantitatively with a given biological activity. Therefore, the activity of compounds with unknown activity can be inferred relying on the combination of the molecular descriptors they show. However, the purely dynamic aspects such as the receptor structure and nature and the ligand-receptor binding event can be only partially derived from the structure of ligands. Conversely, the structure-based approaches rely on the three-dimensional information of the receptors structure. These methods are typically based on the computation of the ligand-receptor interaction allowing a direct analysis of the very early step of MIE. This molecular event is commonly calculated at an atomic level computing the contribution of each atom–atom interaction (e.g., [40,41]). Such approaches are particularly valuable to analyze non-genotoxic chemicals relying on the assumption that a necessary, albeit not sufficient, condition to trigger an adverse effect is the interaction with specific biological targets.

In the framework of mycotoxins risk assessment, investigating the toxicodynamics using in silico methods, even in combination with in vitro and/or in vivo data, may support the hazard identification and hazard characterization steps (*vide infra*). Keeping in mind that most of the mycotoxins analogues and metabolites are still poorly available on the market, assessing systematically the activity of the molecules possibly occurring at the site of action is often hardly affordable. However, the use of computational methods may effectively extend analysis over those compounds that cannot be easily sourced (e.g., mycotoxins metabolites and analogues with excessive costs of synthesis or purification). In addition, molecular modeling may straightforwardly account a broad range of biological targets, giving the chance to include also systems not easily investigable using canonical experimental trials (e.g., specific mutated forms of the biological targets of interest or proteins particularly challenging to be expressed and purified). In the section below, some of the forefront challenges in assessing the toxicology of mycotoxins have been reported, along with discussing the potential role of the in silico molecular analysis in moving the current knowledge ahead.

### 3.1. Computational Tools in Assessing the Combined Toxicity

Although it is still very challenging, assessing systematically the combined effects of mycotoxins and metabolites can be of great relevance for understanding the real role that these compounds may have in living organisms. However, so far, the assessment of combined toxicity has been limited to few parental compounds (e.g., [42,43,44]), although different mycotoxins and many more toxic analogues and metabolites can be found together [45,46,47]. The criteria adopted in selecting the compounds to test are often based on arbitrary principles mostly depending on the practical affordability of compounds. In addition, combinations of the human metabolites formed upon ingestion that likely occur at the site of action are seldom assessed. This scenario strongly suffers from the lack of a common framework providing objective criteria to hierarchize those compounds worthy to be tested together. Concerning the identification of mycotoxins with common mechanisms of action, computational analysis, and in particular the procedures referred to as virtual screening, may be effective in supporting the analysis of the combined toxicity.

Virtual screening procedures refer to as the computational workflows used to search extensive libraries of compounds to identify molecules which are most likely to have pharmacological/toxicological effects of interest [48]. They have been adopted for many years in drug discovery [49], and more recently in food toxicology (e.g., [50]), to find molecules with specific biological properties. In the context of mycotoxins toxicology, all the mycotoxins and metabolites that may co-occur in food and/or in living organisms at the site of action could be affordably screened in silico for the capability to interact with a given receptor. Both the ligand- and structure-based approaches can be effectively used for this purpose. The former derive the capability of molecules to interact with the receptor on the basis of to what extent they deviate from the optimum in terms of the molecular descriptors sorting. The latter provide direct evaluations of the ligand-receptor interaction computing the binding architecture and estimating the energetic favors of the mycotoxins-receptor complex formation.

On this basis, virtual screening procedures may provide objective prioritizing criteria to select those candidates that deserve attention for further investigations, focusing thus the efforts in terms of compounds sourcing and/or synthesis. In addition, they could offer a time and cost-effective tool as a first step toward the comprehensive elucidation of the toxic equivalents of mycotoxins. In this direction, a hybrid in silico/in vitro work has been done by Ehrlich and co-workers expanding knowledge on the (xeno)estrogenic activity of zearalenone group members [51]. Zearalenone may undergo in living organisms reducing and oxidizing metabolism [52,53,54] and many reduced and oxidized metabolites likely occur together at the site of action. However, while the reduced metabolites (e.g., alpha and beta isomers of zearalenol and zearalanol) have been largely investigated for the capability to bind and activate the estrogen receptors (e.g., [55,56]), very few data have been collected for the oxidized metabolites (e.g., 13- and 15-hydroxy-zearalenone and the alpha and beta isomers of 6- and 8-hydroxy-zearalenone). Specifically, the molecular modeling procedure proved to be reliable in estimating quantitatively the estrogenic activity of zearalenone group and so the analysis has been extended on the set of oxidized metabolites. These compounds were found to raise a lower estrogenic concern in comparison to the reduced forms because the hydroxylation patterns was found not complying with the hydrophobic environment of the pocket, causing therefore a reduced ligand-receptor fitting. As exception, 15-hydroxy-zearalenone was identified as a candidate worthy of additional high-priority investigations. This was due to the additional hydroxyl group, potentially able to strengthen the receptor-ligand interaction in comparison to zearalenone. Nonetheless, such interaction was found dependent on the pH as both zearalenone and its 15-hydroxy metabolite may undergo deprotonation equilibrium on the phenolic moiety. Only the neutral forms were actually found able to interact with the estrogen receptors. However, under physiological conditions (i.e., at pH 7.4) the neutral form of 15-hydroxy-zearalenone was predicted less abundant than the parent compound, in agreement with the actual reduced activity of 15-hydroxy-zearalenone experimentally proved by Drzymala and co-worker later on [57].

### 3.2. Computational Tools in Assessing the (Poly)toxicology of Mycotoxins Action

From a molecular perspective, any biological activity, including toxicity, mirrors changes at the level of integrity and functions of DNA, RNA, proteins, and/or small molecules. As aforementioned, the MIE of most of these changes are due to ligand-receptor interactions. However, some mycotoxins show pleiotropic action, acting simultaneously at multiple levels and interacting with more than one receptor [6]. As an example, alternariol (a mycotoxin produced by *Alternaria* species) may act as xenoestrogen (e.g., interacting with the estrogen receptors [58]), as full agonist for androgen receptor [59], and as DNA-damaging agent (e.g., poisoning the topoisomerases enzymes [60,61]). It is becoming increasingly evident that mycotoxins, like drugs and other pharmacologically active molecules, tend to interact with more than one target receptor and signaling pathway. For drugs, this phenomenon is referred to as polypharmacology [62] and analogously from here on it is referred to as (poly)toxicology in the case of mycotoxins. The (poly)toxicology is still poorly understood and investigated for food-related compounds. As a matter of fact, the evaluation of the toxic potential of mycotoxins is based on data concerning the commonly accepted endpoint, neglecting in the most cases to consider any eventual off-target activity. As an example, the class of zearalenone includes a series of well-characterized estrogenic compounds causing severe endocrine disrupting effects in living organisms [63,64]. From a molecular perspective, the best characterized MIE of zearalenone estrogenicity has been related to the capability to bind and activate the estrogen receptors [65]. Accordingly, the relative toxic potency of zearalenone metabolites and analogues has been derived on the basis of the respective suitability in triggering this specific MIE.

The estrogen-dependent signaling pathway encompasses many interconnected steps, with possible interaction with other proteins involved in the pathway. However, the activity of zearalenone and modified forms on the other receptors along the endocrine axis has been poorly characterized so far.

In the past years, many strategies have been developed in medicinal chemistry to identify unexpected targets of pharmacologically active compounds. In this framework, there is a huge number of techniques commonly referred to as “target fishing” that rely on proteomic-, genomic- or bioinformatics-based methods [66,67]. The use of bioinformatic approaches usually has the advantage of being more straightforward and cost-effective in the wide-scale. In more detail, target fishing is widely used in drug design and development as the preliminary step of drug repositioning (i.e., the target switching of a given drug), or in the identification of the off-targets (i.e., the proteins erroneously targeted by a given drug) [68,69]. However, the same workflow could be effectively used to discover further target receptors of mycotoxins moving forward the mechanistic understanding of toxic action. This could strongly support the hazard identification and characterization process in targeting endpoints of real concerns of emerging mycotoxins, being their MIE and/or MOE unknown in many cases.

In the framework of identifying specific receptors for mycotoxins, computational studies have been done on ergot alkaloids. Ergot alkaloids are tryptophan-derived toxins contained in the sclerotia of *Claviceps* spp. [70]. They may accumulate in cereal-based products severely threateninghuman and animal health due to the effects on the circulatory and nervous systems [71,72]. From a toxicodynamics point of view, it is known that the serotonine receptors are relevant biological targets [73], but the relative affinity of the human metabolites toward the different serotonine receptor isoforms has not been elucidated yet. In this framework, the effects of human metabolism in modulating the interaction of ergotamine with the various receptor isoforms (namely, 5HT-2A, 5TH-2B and 5HT-2C) have been investigated in a molecular modeling study [74]. In particular, the hydroxylated ergotamine’s metabolites were found able to interact with the receptors, in agreement with experimental evidences, but with a preferential binding with the 5HT-2A. Conversely, all the conjugated phase-II metabolites were predicted as unable to interact with the receptors considered, suggesting a strong role of phase-II metabolism in impairing the toxicodynamics of ergot alkaloids.

A similar approach has been used by Ivanova and Spiteller [75,76] on several functionalized ergot alkaloids to define structure–activity relationships and calculate the interaction with 5-HT2A, α1/α2-adreno- and dopamine D1–D3 receptors. The authors found that many modified forms might interact with more than one target, providing precious information to understand the biological properties of the compounds analyzed.

### 3.3. Computational Tools to Understand Interspecies Variability

As reported before (Section 2), the risk assessment of food-related contaminants is based on the extrapolations from toxicological data obtained mainly from in vivo animal experiments. However, data extrapolation ideally requires: (i) Detailed information on the mechanisms of toxic action, (ii) defining the species-specific relationship between the MIE and the final adverse outcome [77].

There are many factors affecting the interspecies variability, and both dynamic and kinetic aspects may be involved. From the kinetic point of view, interspecies differences may be due to multiple differences along the entire ADME pathway. As examples, the differential species-specific absorption of mycotoxins has been proved (e.g., [78]). Also, keeping in mind that metabolism may also drive to a bioactivation of the parent compound, different species may produce different toxic/detoxified metabolites ratio. As examples, this is involved in determining the interspecies susceptibility to zearalenone. In particular, it has been found that the species susceptible to zearalenone (e.g., pig) prevalently produce the more toxic alpha isomers of the reduced metabolites zearalanol and zearalenol. Conversely, the resistant species are strong beta-converter with the beta isoforms prevalent over the alpha (e.g., in poultry [79]). Concerning the dynamic aspects, differences may be due to differences in the biological target sequences due to the speciation event that affects the recognition of mycotoxins (see below). Therefore, understanding the toxicodynamic basis of interspecies variability may promote a more informed extrapolation of data. In this framework, computational analysis can provide direct analytical tools to investigate the mechanistic basis of inter-species differences.

Orthologous proteins typically maintain the overall organization along the speciation pathway as it is mandatory for keeping the specific function among the species [80]. Rather, orthologous proteins usually differ in those regions devoted to the fine modulation of proteins function. Also, differences in these regions can be causally connected to the different interspecies susceptibility [81]. These structural differences may imply extended modification of the quaternary structure of proteins, such as the addition or deletion of regulatory domains, or minor modifications in terms of amino acid deletion, insertion or substitution [82,83,84]. It may happen that these modifications occur at the level of binding site or in the regions modulating the path for accessing or leaving the binding site (see below).

**Figure 1:**
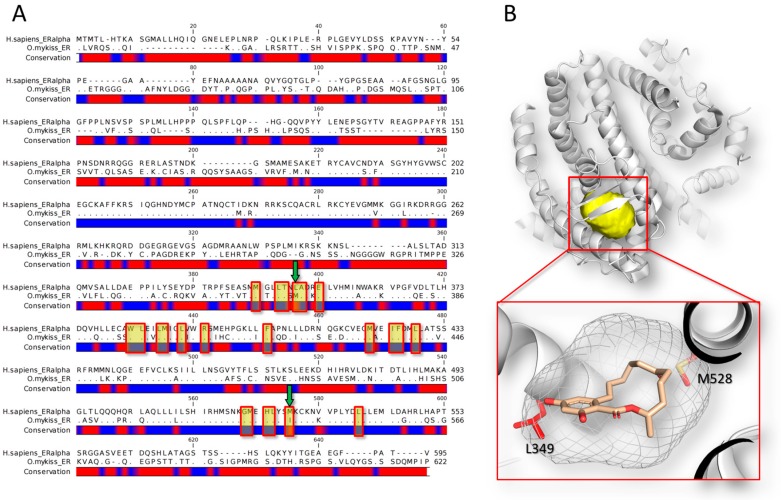
Sequence and structural analysis of the human estrogen receptor alpha (*Homo sapiens;* GeneBank Accession number: P03372.2) in comparison to the receptor of rainbow trout (*Oncorhynchus mykiss*; GeneBank Accession number: NP_001117821.1). (**A**) Sequence alignment of human (line above) and fish (line below) receptors. The alignment was done using the CLC Sequence Viewer software version 7.7 (QIAGEN, Aarhus, Denmark; https://www.qiagenbioinformatics.com). Dots represent matching residues while dashes indicate gaps. The red spots in the conservation bar indicate the non-conserved regions. The residues forming the binding site are highlighted in yellow, while green arrows indicate the non-conserved residues in the binding site; (**B**) 3D representation of the human estrogen receptor dimer in complex with zearalenone [85]. The pictures have been obtained using the software PyMol version 1.7 (Schrödinger, New York, NY, USA; http://www.pymol.org). The protein (human estrogen receptor alpha; PDB code: 5KRC [85]) is represented in cartoon while the binding site is represented in yellow surface. The close-up in the red box shows the binding pose of zearalenone (represented in stick). The shape of the binding site is represented in white mesh, while the non-conserved residues in the fish orthologous (i.e., L349 and M528, according to the human numeration) are represented in red stick.

In this regard, Matthews and co-workers [56] showed that zearalenone and its metabolite α-zearalenol bind the estrogen receptors of rainbow trout with a stronger affinity than that observed for the other species tested (i.e., human, mouse, chicken and green anole). The sequence analysis shows that the human and fish orthologous have 46.5% of identity in terms of amino acid sequence conservation (Needleman–Wunsch alignment algorithm) (Figure 1). The non-conserved region includes also two mutations at the level of the binding site (L349M and M528I, according to the human numerations). Overall, all these differences, including those at the level of the binding site, concur to differentiate the interspecies binding activity, eventually leading to a different magnitude in triggering the xenoestrogenic action.

The precise understanding of what determines differences among species at the very beginning of the MIE may expand the comprehension of the mechanisms of action providing meaningful information to ameliorate the data extrapolation. In this view, computational analysis, and in particular the structure-based approaches, may investigate the primal molecular event underlying toxic action from an atomistic point of view [86] providing the structural rationale of such differences. Even though no data are still available in this direction for mycotoxins, a more and more growing number of proteins structures from a huge spectrum of organisms are going to be available in public repositories (e.g., the Protein Data Bank; https://www.rcsb.org [87]) making theoretically affordable the inter-species comparison of the mode of binding of mycotoxins.

### 3.4. Computational Tools in Accounting Inter-Individual Variability: A First Step in Personalized Risk Assessment?

In the recent years, many efforts have been done to focus medical and healthcare interventions on the individual or small groups of individuals to provide more specific and effective actions. In this framework, it has been developed the principle of personalized risk assessment that is referred to as the process where an individual’s level of risk is calculated using multiple predictors that are specific for an individual [88]. Such an approach is getting more and more attention as similar doses of specific xenobiotics may cause different responses not only among different species, but also among different individuals of the same species (e.g., [89]). Additionally, the presence of pathologic conditions may cause a more pronounced susceptibility making the personalized intervention even more needed for certain sub-population groups. The susceptibility differences have been attributed mainly to genetic, epigenetic and environmental factors [90]. Additionally, even though assessing the individual risk requires holistic approaches, toxicodynamic studies may concur to better understand the molecular basis of individual response. Currently, the inter-individual toxicodynamic variability is addressed using AOP-based approaches (e.g., [91]). Accordingly, it is assumed that compounds sharing the same receptor and the same physiological effects might also show the same extent of inter-individual variability in toxicodynamics [92]. However, in the case of genetically-induced variability related to the expression of mutated forms of specific proteins, toxicodynamic aspects may be directly investigated focusing on the differences at the level of toxicant-receptor binding. Indeed, mutations may occur on toxicologically relevant receptors affecting in turn the dynamic of the receptor-toxicant complex formation. As an example, some recurring mutations at the level of the estrogen receptors binding site have been found in estrogen-dependent cancer [93]. Some mutations are related to the abnormal constitutive activation of the estrogen receptors giving resistance to the competitive anti-estrogen inhibitors 4-hydroxytamoxifen and fulvestrant by affecting their capability to interact with the estrogen receptor binding site [94].

Concerning mycotoxins, investigating the personalized response of specific subpopulation groups or individuals is still a largely overlooked field of research. It has been proved that some population groups may be more exposed to mycotoxins than others depending on environmental factors, diet habits or alimentary regimes [15,95]. In addition, sensitive categories may be defined depending on the developmental stage of individuals taking into account diet restrictions and the physiological differences in the metabolic rate and in the ability to detoxify food toxicants. For these categories, e.g., infants and young children, specific regulation may exist [96]. However, assessing the effects of mycotoxins on subjects bearing specific pathological or physiologically altered conditions is a largely understudied topic. Nevertheless, in analogy with other xenobiotics and drugs (see above), a degree of individual response to mycotoxins exposure cannot be excluded a priori and it might be a further object of investigation moving toward the establishment of more effective personalized healthcare interventions.

As an example, the zearalenone group might be a good candidate to be analyzed from a toxicodynamic point of view on account of the: (i) Controversial role of zearalenone in breast cancer progression and maintenance [97]; (ii) involvement of estrogen receptor mutations in breast cancer cell growth; (iii) the role of estrogen receptor mutations in altering the ligand-dependent activation of the estrogen receptors (see above). Therefore, it might be worthy assessing the potential effects of zearalenone group members on such mutated forms, detailing the molecular basis of eventual differences. Evidences in this direction have not been collected yet, but there is no data ruling out a possible relevance to humans. Thus, the scientific acceptance will require rigorous investigations. In this framework, computational models may be straightforward analytical tools as they may succeed in estimating the effects of mutations in receptor functionality and ligand binding [98,99,100]. Therefore, even though not still implemented, in silico modeling might be a first-line analytical tool supportive of the mechanistic analysis of intraspecific variability of mycotoxins.

## 4. Case Studies

As aforementioned, the understanding of mechanistic basis of most of the mycotoxins action is still in its infancy. However, for some mycotoxins, it has been possible to draw a sound background of knowledge merging together in silico results with experimental evidences. Some of the best characterized case studies are reported below.

### 4.1. Estrogenic Activity of Zearalenone Group

The zearalenone group perhaps is one of the best-characterized classes of mycotoxins from the toxicodynamic point of view. Zearalenone is a mycotoxin produced by fungi belonging to *Fusarium* spp., mainly *F. culmorum* and *F. graminearum*, which may accumulate in small grains, maize and derived products [101]. In addition to the attested cytotoxic and genotoxic effects, zearalenone group poses health risks for humans and animals on account of the (xeno)estrogenic activity [102]. Typically, zearalenone co-occurs in food and feed with a number of fungal analogues (e.g., the alpha and beta isomers of zearalanol and zearalenol) and plant metabolites (e.g., zearalenone-glycosides and sulfates) [103]. From a molecular point of view, the capability to trigger the (xeno)estrogenic stimulus has been considered mainly related to the capability to bind and stabilize the estrogen receptors in the so defined agonistic conformation [51]. Many works in the last years assessed the structure-activity relationship of zearalenone analogues identifying the alpha isomerism of the reduced metabolites zearalenol and zearalanol as relevant for enhancing the capability of binding and activating the estrogen receptors [104]. Recently, such differences have been elucidated from toxicodynamic perspective. Indeed, zearalenone and the reduced metabolites isomers have been found able to well-fit the receptor pocket, as showed by in silico modeling studies [51,65] further confirmed by crystallographic evidences [85,105]. However, the hydroxyl group in the alpha isomerism was found engaging the receptor pocket with an additional hydrogen bond, strengthening therefore the overall receptor-ligand interaction in comparison to zearalenone. Concerning the conjugated forms originated by plant metabolism (referred to as masked mycotoxins) [106], they can be a relevant part of the total toxicants load and, in some cases, they can be even more abundant that the respective parent compound [107,108,109]). Accordingly, they are supposed to have a not-negligible role in determining the overall group toxicity. However, so far, very little is known about the toxicity of the masked forms, even though some general properties can be inferred on the basis of the data available. The best-characterized masked form is zearalenone-14-glucoside. Its (xeno)estrogenic activity has been disputed for a long time, along with the supposed underlying mechanisms of action. In fact, this compound *per se* was found unable to bind the estrogen receptors in a competitive binding assay [110], while a substantial estrogenic activity has been observed in cell-based assays [111]. The combination of in silico data and in vitro evidences provided insights on the mechanistic basis of these apparently conflicting data. In particular, molecular modeling studies have shown the incapability of zearalenone-14-glucoside to fit within the pocket of the estrogen receptors in the active conformation [111]. The capability to bind the estrogen receptor stabilizing the so defined agonist conformation is the *sine qua non* condition for assembling the transcriptional machinery and transducing the toxic action [112]. Steric hindrance and hydrophobic/polar mismatches due to the posing of the glycoside moiety into a spatially-constrained and hydrophobic environment have been identified as the structural basis of such unfavored interaction. On this basis, zearalenone-14-glucoside *per se* proved to be inherently unable to trigger or propagate the molecular events of MIE. However, zearalenone-14-glucoside was found undergoing hydrolysis to zearalenone and other estrogenically active metabolites in the experimental conditions commonly used to assess the mycotoxin estrogenicity [113]. In particular, fetal bovine serum and bovine serum albumin (that were both growing-medium components in the cell-based assay) have been found involved in the hydrolysis reaction. Taken together, these results suggested that zearalenone-14-glucoside is unable to act *per se* as estrogenically active compound. Furthermore, on the basis of the pocket requirements highlighted by the computational analysis and structural evidences [51,85,105], any conjugated form is expected as unable to fit the agonistic estrogen receptors pocket. This evidence has been further confirmed by proofs on glucuronidated metabolites stating the lack of a substantial estrogenic activity *per se* [114]. Rather, masked mycotoxins should be carefully accounted assessing eventual hydrolysis phenomena able to release the parent form and/or other known estrogenically active metabolites. Therefore, considering the results collected so far concerning the zearalenone group, it can be inferred that the reduced forms, and in particular the alpha isomerism, are those of most concern from a toxicodynamic point of view. Accordingly, they may have a pivotal role in determining the number of toxic equivalents at the site of action. However, it shall be needed to check the actual abundance of the various forms at the site of action to further deepen the dynamic aspects of zearalenone group.

### 4.2. Topoimoerase I Poisoning by Alternariol

Alternariol belongs to the so defined emerging mycotoxins [115] and it is mainly produced by *Alternaria* spp. *Alternaria* toxins may accumulate in many food types, but cereals and related products, tomato and sauces, sunflower seeds and oil, fruits, beer and wine are the main source in the human diet [116]. Although these mycotoxins are not still regulated, they should be accounted in risk assessment studies due to their toxic activity. As examples, alternariol has been found acting as potential (xeno)estrogen [58], as full agonist for androgen receptors [59] and as DNA-damaging agent [117] via the poisoning of topoisomerases enzymes [60]. In addition, the contamination by *Alternaria* mycotoxins has been supposed to be involved in the formation of esophageal cancer [118]. From a mechanistic point of view, the group toxicity of *Alternaria* mycotoxins in terms of estrogenic and androgenic activity is still poorly characterized. Conversely, Dellafiora and co-workers [119] provided molecular insights on the topoisomerases inhibition focusing on the topoisomerase I. In particular, they used a step-wise computational approach coupling ligand-based virtual screening and molecular modeling to elucidate the structural basis of the activity found experimentally. Then, the analysis was extended to a series of metabolites with unknown activity that may occur at the site of action, to identify further forms possibly involved in determining the overall number of toxic equivalents.

**Figure 2:**
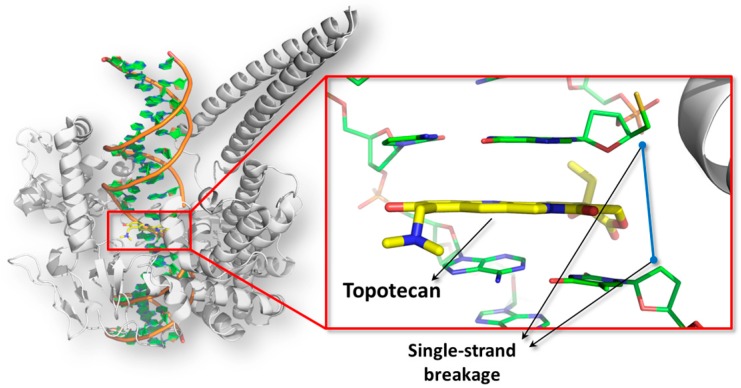
The poisoned topoisomerase I-DNA complex is shown. The picture has been obtained using the software PyMol version 1.7 (Schrödinger, New York, NY, USA; http://www.pymol.org). The structure of the human topoisomerase is represented in white cartoon, while the DNA double helix is colored in orange (PDB code: 1K4T [121]). In the close-up, the intercalating event of the topoisomerase I poison topotecan (shown in yellow sticks) is reported.

With more details, topoisomerase enzymes form cross-link with DNA introducing single or double strand breakage to relax the supercoiled state of chromatin. At the end of the reaction, the strand breakages are resealed and the enzymes are set free for further reactions. The poisoning is a particular type of inhibition mechanism wherein small molecules prevent the resealing event intercalating the DNA double helix at the catalytic site and converting the enzyme in a DNA-damaging agent [120] (Figure 2).

Computational analysis revealed that the binding site appears as a lock-shaped cavity formed by both DNA and amino acids. The inner pocket environment was found prevalently hydrophobic and surrounded by a fairly extended polar space mainly energetically favorable to receive hydrogen-bond donor groups. Besides alternariol, also altenuene, iso-altenuene and alternariol methyl ether have been tested for topoisomerases poisoning, but only the former was found active toward the topoisomerase I up to 100 µM [60]. Computational analysis revealed that the aromatic moiety of alternariol drove the π-π stacking needed to interact with the ligand site, while the hydroxyl group in position #3 was found engaged in polar interaction with the enzyme. Conversely, the lack or aromaticity in altenuene, iso-altenuene, and the presence of an additional methyl group of alternariol methyl ether were found interfering with π-π stacking providing a mechanistic basis of the inactivity found experimentally. Concerning the extended analysis on the other metabolites, a number of molecules originated from phase-I and phase-II metabolism were found possibly concurring to the overall number of toxic equivalents as they were found able to fit the receptor site. Among them, some glucuronidated metabolites were found potentially active. They draw particular attention on account of the few epidemiological data available so far which suggest an involvement of Alternaria toxins in the etiology of esophageal cancer [118,122]. In particular, topoisomerases poisoning causes DNA instability and chromosomes breakage, which are hallmarks of cancer cells, and topoisomerases alteration have been found in cancer cells as well [123,124,125]. Therefore, taking into account that esophageal tissue particularly expresses several UDP-glucuronosyltransferases [126], such glucuronides have been identified in the framework of hazard identification as potential hazards to be tested further as possibly involved in esophageal carcinogenesis.

### 4.3. (Poly)toxicology of Ochratoxin A

Ochratoxin A is a mycotoxin produced mainly by *Aspergillus* and *Penicillium* spp. that may accumulate in a huge number of food including cereal products, olives, beans, beer, wine, coffee, cocoa products, raisins, figs, licorice, pulses, pumpkin seeds, and tea [127]. It has been linked to the so defined fatal human kidney disease “Balkan endemic nephropathy” [128], and it has been historically regarded as a potent renal carcinogen in rats, with a clearly more pronounced susceptibility of male animals [129]. Moreover, in addition to nephrotoxicity, it has been found to exert hepatotoxicity, and a degree of mutagenic activity has been reported as well [130]. The International Agency for Research of Cancer (IARC) classified ochratoxin A as a possible carcinogen for humans (group 2B) [131]. The mechanisms of carcinogenic action are not still fully understood yet [132]. However, along with the DNA damage due to an enhanced production of reactive species of oxygen and nitrogen, it has been hypothesized a prominent role of its direct genotoxic properties via the formation of DNA-adducts [133,134]. The direct ability to forms adducts may require metabolic activation by cytochromes and/or peroxidase enzymes activity [134,135]. Structure-activity relationship studies revealed that the C5 chlorine atom primarily affects the chemical reactivity to form adducts, while it affects to a lower extent the cytotoxic activity [136].

From a molecular point of view, five key steps have been found acting in the renal tumor formation: toxin uptake at the level of proximal tube epithelium, inhibition of histone acetyltransferases, disruption of mitosis, cell proliferation and genetic instability [137]. In particular, it has been hypothesized that the pronounced renal susceptibility is due to active transporters at the level of proximal tube epithelium that facilitate the transport of ochratoxin A into the kidney cells [138]. The prevalent expression of the toxins transporters in male in comparison to female may explain the gender-dependent effects found [137,139]. In addition, ochratoxin A is thought to alter a cascade of molecular events via inhibition of histone acetyltransferases [132], which may cause, *inter alia,* alterations in the cell-cycles progression during mitosis. This may lead to apoptotic death or to the generation of genetically unstable polyploid cells that may re-enter the cell cycle [137].

Taken together, these results clearly show that both specific mechanisms involving receptors binding and genetic and environmental factor are involved. More germane to the toxicodynamic aspects, it has been found that many biological targets are involved in mediating the ochratoxin A toxic action, including enzymes and carriers (see above), thus sustaining its broad (poly)toxicology action. Concerning the interaction with specific enzymes, two of the best characterized ochratoxin A targets are the enzymes Phe-tRNA synthase and Phe hydrolase [140]. In particular, the interaction with the former has been investigated from a molecular point of view revealing the structural basis of the toxin-enzyme interaction [141]. The interaction with the phenylalanine-related enzymes have been thought to be mainly mediated by the phenylalanine moiety. In particular, ochratoxin A was thought arranging the phenylalanine moiety in the buried side of the enzyme pocket analogously to the mode of binding of the natural enzyme substrate phenylalanyl adenylate and its inhibitory analogues [142]. However, the computational studies highlighted the inability of ochratoxin A to overlap the Phe-moiety of phenylalanyl adenylate. Indeed, the buried side of the enzyme pockets was found favorably occupied by the coumarin moiety with the Phe-moiety engaged in polar interactions with residues accessible to the bulk water [141,143]. The authors provided for the first time structural evidences on the receptor binding of ochratoxin A, pointing out the toxicological relevance of the coumarin moiety in the mechanisms of action. The proposed not-exclusive role of the Phe-part in inhibiting aminoacyl-tRNA synthase is in agreement with studies showing that modified ochratoxin A analogues with other amino acid substituents may keep inhibitory activity [144]. At the same time, the not-sufficient role of the coumarin part alone has been proved by the lack of activity found for the metabolite ochratoxin-alpha, which lacks the Phe-moiety [144]. Nevertheless, recent insights on the structure-activity relationship of ochratoxin A and analogues have showed how substitution of the Phe-part or the chlorine atom may prevent all the adverse effects of ochratoxin A in human renal cells [145]. Taken altogether, these results point out that the Phe-moiety might play a major role in the interactions with some other key targets involved in mediating the toxic action (e.g., the carriers involved in the cellular uptake).

In this respect, it is worth mentioning the interaction with the human serum albumin. Two binding sites have been identified for ochratoxin A with binding constants in the range of drug-like compounds (from 10^−4^–10^−7^ M) and differing from each other by a factor of 50 (the one with the high affinity is in the domain IIA, the one with the low affinity in the domain III) [146]. Mutagenesis studies revealed that the residues Arg218 and Arg257 are important for the toxin binding and orientation. In particular, Arg218 is thought to be involved in the stabilization of the dianion form that occurs in the physiological condition, but not in determining the overall binding geometry. Conversely, Arg257 has been found affecting both the binding affinity and geometry of interaction as it is thought to interact and orient the lactone portion of ochratoxin A [146,147]. Evidences collected so far have suggested that understanding the dynamic of interaction with the serum albumin may be relevant for understanding both toxicodynamic and toxicokinetic aspects of ochratoxin A in vivo. Indeed, it has been proved that the presence of human serum albumin (0.025% *w*/*v*) may almost completely abolish the uptake of toxin by *Xenopus laevis* oocytes [148]. Therefore, albumin binding may impact the transport in vivo and thus it must be considered when accounting both the toxin clearance and bioaccumulation [146].

### 4.4. Ribotoxicity of Trichothecenes

**Figure 3:**
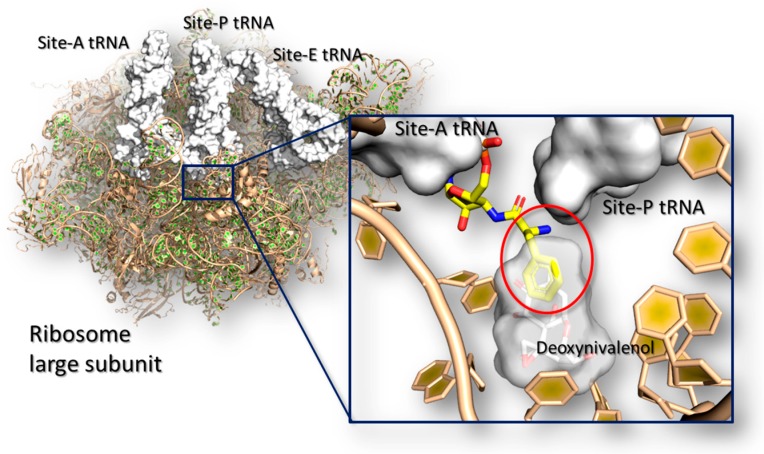
The mechanism of ribosome inhibition by trichothecenes is shown. The pictures have been obtained using the software PyMol version 1.7 (Schrödinger, New York, NY, USA; http://www.pymol.org). The structure of the ribosome large subunit is represented in cartoon (PDB code: 4U53 [155]) while tRNAs are represented in white surface (PDB code: 4V5C [156]). In the close-up, the sterical clashes between deoxynivalenol (represented in white surface and stick) and the Phe-tRNA is shown within the red ring.

Trichothecenes are a huge group of chemically related compounds that are produced mainly by *Fusarium* spp. [149]. They are extremely prevalent worldwide [150] and they may accumulate in a variety of food including wheat, barley, oats, maize and derived products [151]. Among this class, deoxynivalenol is one of the most widely occurring mycotoxins, and it may cause severe injuries in human and animals [152] with an evident low-tolerability in mono-gastric animals [153]. In living organisms, trichothecenes elicit a wide number of effects including dysfunctions at the gastro-intestinal apparatus, alteration in the normal food intake, nutrients absorption, immunosuppression and susceptibility to infections [152]. All these generalized effects are caused by a complex network of molecular events that likely involves the interaction of mycotoxins with many target receptors. However, most of them are still unknown blurring the overall comprehension of the trichothecenes toxicodynamics. Nonetheless, from a molecular point of view, it is known that deoxynivalenol and other trichothecenes (e.g., T-2 toxins and verrucarin A) inhibit protein synthesis by interfering directly with the ribosome and causing the so defined ribotoxic stress [154]). In particular, crystallographic studies revealed that trichothecenes may bind the A-site of the ribosome large sub-unit at the catalytic site, where they impair the elongation of the growing peptide chain interfering with the tRNA accommodation [155] (Figure 3).

Some deoxynivalenol analogues have been assessed for the capability to bind and inhibit the ribosome. Among them, 15-acetyl-deoxynivalenol, T-2 toxin and verrucarin A proved to be able to bind ribosome site similarly or more strongly than deoxynivalenol [152,155]. Conversely, deepoxynivalenol, deoxynivalenol-15-sulfate and all the forms with modification in position #3, including 3-acetyl-deoxynivalenol, the epimeric form 3-*epi*-deoxynivalenol, deoxynivalenol-3-sulfate and deoxynivalenol-3-glucoside, were found substantially less toxic than deoxynivalenol [152,157,158]. It is worth noticing that computational studies succeeded in identifying the site of binding in the lack of crystallographic evidences [159] and further molecular models revealed the structural basis of such differences observed experimentally. In particular, Pierron and co-workers [157] showed that deepoxynivalenol and 3-*epi*-deoxynivalenol are less toxic than deoxynivalenol. Then, they used a combined in vitro/in silico approach for analyzing the molecular basis, pointing out that the differences in toxicity may be due to changes in the ligand-receptor interaction network. Specifically, the deoxynivalenol was found forming additional polar interactions with the ribosome site in comparison to deepoxynivalenol and 3-*epi*-deoxynivalenol, leading to a stronger receptor binding and inhibition power.

These results have been confirmed by a further molecular modeling study by Dellafiora and co-workers [160]. The authors extended the analysis to a broader set of molecules providing a more comprehensive structure-activity relationship analysis on the trichothecenes class. In particular, they showed that the position #3 is critical for impairing the capability to interact with the ribosome mainly due to steric hindrance. As an example, the lack of activity of deoxynivalenol-3-glucoside and 3-acetyl-deoxynivalenol found experimentally [152,161] was explained on the basis of sterical clashes with the Mg^2+^ ion that prevents the arrangement of deoxynivalenol modified forms with bulky groups in this position. The authors highlighted that many forms arising from plant and human metabolism (including phase II metabolites) may well fit the receptor site.

## 5. Conclusions

The risk assessment of mycotoxins and the advances in their toxicological understanding are tightly related. Indeed, only a few of food-related mycotoxins have been accounted in risk assessment studies and most of them are still lacking a satisfying toxicological characterization, especially from a mechanistic point of view. Nevertheless, further insights on the mycotoxins toxicology are needed to promote more effective risk assessment studies.

**Figure 4:**
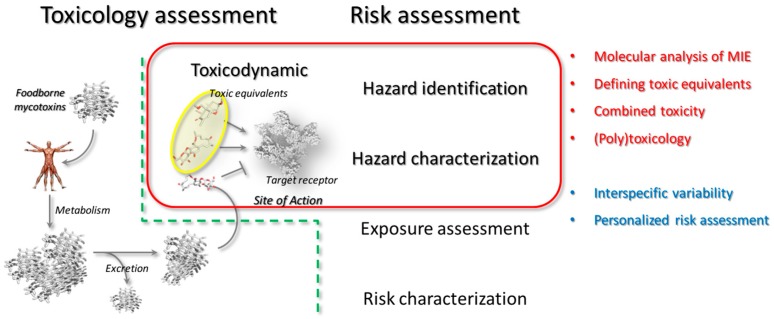
The interconnection between mycotoxins toxicology and the risk assessment process is shown. In particular, the support of the in silico analysis in the hazard identification and hazard characterization processes has been summarized in red, while possible future applications have been indicated in blue.

Investigating mycotoxins toxicology poses inherent challenges that are primarily depending on the poor availability of compounds. Keeping in mind that deciphering mycotoxins action inevitably requires inter-disciplinary approaches and both kinetic and dynamic aspects need to be considered, in silico models can be affordable fist-line analytical tools to support the in vitro and/or in vivo toxicological investigation. In particular, even though computational/mathematical approaches may succeed in modeling toxicodynamic/toxicokinetic aspects [33], the present review covers the methods addressing the early mechanistic steps of toxic action. Some of the possible applications in the framework of hazard identification and hazard characterization have been presented, as summarized in the Figure 4.

Specifically, molecular modeling approaches may be upstream and cost-effective analytical tools in the identification of those mycotoxins and/or metabolites competing for a specific receptor, thereby showing a potential combined action. In addition, the use of the so defined “target fishing strategies” may also concur to define and discover relevant receptors potentially targeted by a specific mycotoxin or mycotoxins group(s). This might be relevant especially for the emerging mycotoxins as in many cases they have still unknown MIE and/or MOE.

From a general point of view, molecular modeling approaches may directly account the mycotoxins-receptor complex formation deepening the structural basis of mycotoxins interaction. Additionally, the computation of mycotoxin-receptor interaction may contribute to decipher the basis of the inter- and intra-specific variability.

Therefore, computational studies might be taken into consideration together with experimental evidences for a more informed extrapolation of data from in vitro and in vivo animal studies or to support pioneering steps in the personalized risk assessment of mycotoxins.
